# Bladder pressure-guided delayed abdominal closure in a neonate treated with ECMO for congenital diaphragmatic hernia repair: a case report

**DOI:** 10.3389/fped.2026.1763422

**Published:** 2026-04-09

**Authors:** Bo Xia, Qiu-Ming He, Wei Zhong, Jun-Jian Lv, Qiang Wu

**Affiliations:** Department of Neonatal Surgery Intensive Care Unit, Guangzhou Women and Children’s Medical Center, Guangzhou Medical University, Guangzhou, China

**Keywords:** abdominal compartment syndrome, bladder pressure, case report, congenital diaphragmatic hernia, delayed abdominal closure, ECMO

## Abstract

**Background:**

Delayed abdominal closure (DAC) after congenital diaphragmatic hernia (CDH) repair is often guided by subjective assessment. This is particularly critical in neonates receiving extracorporeal membrane oxygenation (ECMO), where inappropriate closure can precipitate abdominal compartment syndrome (ACS), yet objective guidance is lacking. Here, we describe a case where intraoperative bladder pressure (BP) monitoring provided an objective guide for DAC.

**Case introduction:**

A term male neonate with severe left-sided CDH and pulmonary hypertension required veno-arterial ECMO. On ECMO day 4, he underwent repair of the diaphragmatic defect. Following visceral reduction, abdominal wall tension increased, and BP was measured at 20mmHg. Based on this objective evidence of intra-abdominal hypertension, DAC was performed with placement of a temporary silastic silo. Following decompression, the BP decreased to 8 mmHg. The patient was successfully weaned from ECMO on postoperative day 1. Definitive abdominal wall closure was performed on day 7. The infant recovered and was discharged on day 38 of life.

**Conclusion:**

Intraoperative BP monitoring is a simple, reproducible, and objective tool that can effectively guide the decision for DAC in neonates undergoing ECMO-assisted CDH repair, potentially preventing ACS and improving outcomes.

## Introduction

Congenital diaphragmatic hernia (CDH) is characterized by the herniation of abdominal contents into the thoracic cavity (1). The aim of the surgery is to reduce the herniated viscera and repair the diaphragmatic defect. However, the restricted abdominal space increases the risk of intra-abdominal hypertension and subsequent abdominal compartment syndrome (ACS), which carries a mortality rate of 22∼45% (2–4).

This risk is significantly elevated in neonates treated with extracorporeal membrane oxygenation (ECMO) support. To avoid such complications, delayed abdominal closure (DAC) is often considered. Currently, DAC decisions largely rely on subjective surgical judgment due to the scarcity of objective intra-abdominal hypertension pressure (IAP) data, which is typically obtained via bladder pressure (BP) monitoring in adults and children (4, 5). We present a case in which intraoperative BP monitoring was effectively used to guide DAC objectively in a neonate with CDH undergoing ECMO support, underscoring its importance in preventing ACS and optimizing surgical outcomes.

## Case report

A male neonate with a prenatally diagnosed left-sided CDH was admitted to Department of Surgery Neonatal Intensive Care Unit at Guangzhou Women and Children's Medical Center, Guangzhou Medical University. The transfer was indicated due to progressive respiratory failure refractory to conventional management, necessitating escalation to extracorporeal membrane oxygenation (ECMO) support.

**Figure 1:**
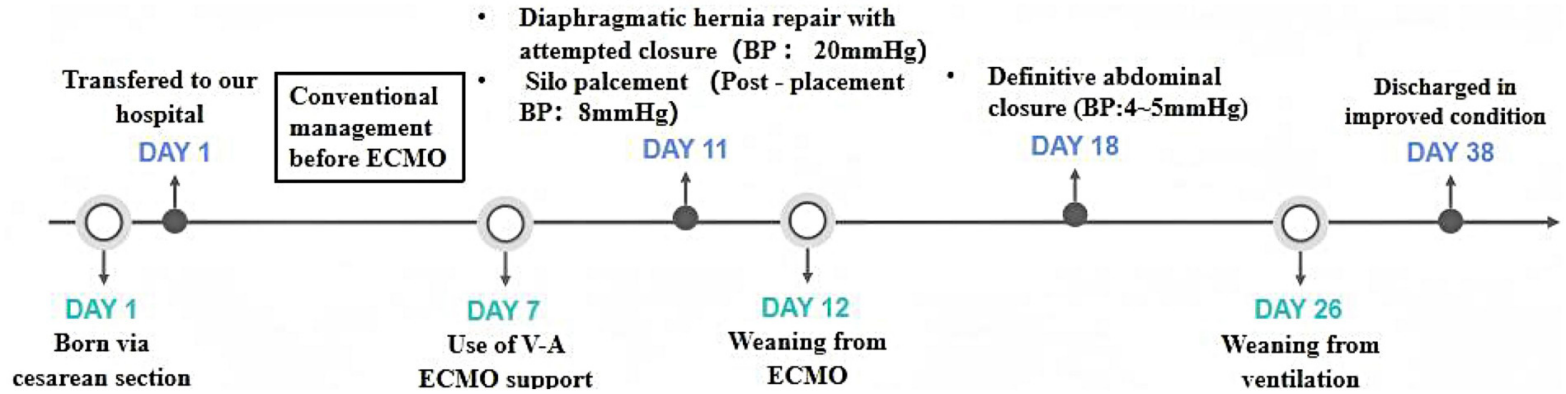
Patient's treatment course.

Routine prenatal ultrasound at 25 weeks of gestation at a local hospital identified a left-sided CDH. Subsequent prenatal assessment suggested moderate-to-severe pulmonary hypoplasia. Fetal MRI performed at 27^+2^ weeks of gestation revealed a total fetal lung volume (TFLV) of 17.91 mL, and ultrasound measurement showed an observed-to-expected lung-to-head ratio (o/e LHR) of 30%. The infant was delivered via cesarean section at 39⁺^2^ weeks, with a birth weight of 3190 g. Apgar scores were 8 and 9 at 1 and 5 min, respectively. Despite immediate intensive cardiorespiratory support at the local hospital, including mechanical ventilation, inhaled nitric oxide (iNO), and multiple vasoactive agents, his clinical condition deteriorated. Due to this critical status, he was transferred to our hospital for further treatment on his first day of life. Figure 1 is a timeline of the clinical condition progress and major management of the patient.

After admission, he developed severe respiratory failure and persistent pulmonary hypertension refractory to conventional management. Echocardiography demonstrated a persistent ductus arteriosus (PDA) with a right to left shunt and associated severe pulmonary hypertension. On the 7th day of life, veno-arterial extracorporeal membrane oxygenation (ECMO) was initiated. While receiving ECMO support with lung-protective ventilation, inotropic support, and systemic anticoagulation, the patient remained hemodynamically stable, and the generalized edema demonstrated significant resolution. He underwent repair of the diaphragmatic hernia via an abdominal approach on the 11th day of life. Intraoperative exploration confirmed herniation of the entire small intestine, most of the colon, the spleen, and the stomach into the thoracic cavity. The diaphragmatic defect measured approximately 3.0 × 2.8 cm (Grade B). Primary abdominal closure was attempted but abandoned due to high intra-abdominal tension, which was confirmed by an intravesical pressure at 20 mmHg via Foley catheter measurement. The intravesical pressure was measured using a standard technique: after insertion of an 8 Fr Foley catheter under aseptic conditions, the bladder was emptied and then instilled with 1 mL/kg (maximum 10 mL) of sterile normal saline to ensure a minimal filling volume for pressure transmission. A pressure transducer connected to the bedside monitor was attached via a three-way stopcock, zeroed at the level of the pubic symphysis with the patient in the supine position, and the pressure was recorded at end-expiration. This elevated pressure was confirmed by a repeat measurement.

Considering this finding and the risks of fluid overload associated with ECMO, a temporary silastic silo was placed to allow for staged abdominal wall closure. Following silo placement, the intravesical pressure promptly decreased to 8 mmHg. Postoperatively, bladder pressure was monitored three times a day; on the first postoperative day, recorded values remained within the range of 4∼7 mmHg.

The patient tolerated the procedure well and was successfully decannulated from ECMO on the first postoperative day. The abdominal domain was managed with the silo, and the patient received aggressive diuresis. As systemic edema resolved and abdominal wall compliance improved, and with bladder pressure stabilized within the normal range of 4∼5 mmHg, definitive abdominal wall closure procedure was performed on postoperative day 7. The infant recovered fully and was discharged home on the 38th day of life. Follow-up at 3 months confirmed satisfactory growth and development with no evidence of hernia recurrence.

## Discussion

This case demonstrates the value of an objective, data-driven approach in neonatal CDH repair. The decision for DAC conventionally relies on the surgeon's subjective assessment of abdominal tension. While clinical experience is invaluable, this judgement can vary and be confounded, particularly in patients on ECMO support. We used intraoperative BP monitoring to obtain a critical and reproducible objective parameter that directly guided clinical management. A value >15 mmHg indicated the patient as high-risk for IAH. Primary closure at this level would likely have induced ACS, adversely affecting respiratory mechanics, cardiac output, and renal perfusion, thereby jeopardizing ECMO weaning and the entire outcome (6, 7).

In adults, the consensus defines IAH as IAP >12 mmHg and ACS as IAP >20 mmHg with new organ dysfunction (8). Notably, for the pediatric population, the WSACS guidelines define IAH as IAP >10 mmHg and ACS as IAP >10 mmHg accompanied by new organ dysfunction (9). However, standardized diagnostic thresholds and monitoring protocols are not well established for neonates, especially those receiving ECMO. In the present case, a BP >15 mmHg was used as the operative threshold to guide the decision for DAC. It is crucial to note that validated, consensus-driven BP cutoff values for defining IAH/ACS in neonates, particularly those on ECMO, are currently lacking. This threshold was pragmatically chosen based on two considerations: First, its alignment with the lower range of Grade 1 IAH (10∼15 mmHg) in pediatrics guidelines (9); and second, our institutional experience and the imperative to avoid any pressure that could compromise perfusion in this high-risk infants. We do not propose that 15 mmHg represents an established neonatal standard, but rather report its utility as an objective intraoperative datum in our specific context. This low-cost, minimally invasive technique is easily performed and holds broad clinical applicability.

The pathophysiology of ACS in patients receiving ECMO support is multifactorial, often involving capillary leak syndrome, significant ascites, bowel edema, and hemorrhage. Coagulopathy and systemic physiological alterations induced by ECMO create an unstable states, where postoperative hemorrhage and visceral edema can acutely elevate IAP. Once ACS develops, it may rapidly initiate a vicious cycle of bowel ischemia, refractory bleeding, and clinical deterioration, substantially increasing mortality risk (10–15). Therefore, vigilant perioperative monitoring for ACS is essential in these infants. Furthermore, diagnosing perfusion disturbances after CDH closure poses a challenge. The effects of elevated IAP and hypoperfusion are not limited to abdominal organs. In cases of large CDH, primary closure under tension can exacerbate IAP, which may secondarily affect cerebral hemodynamics. The proposed mechanism involves a cascade wherein elevated IAP raises intrathoracic pressure, impedes venous return, and reduces cardiac output, ultimately compromising systemic perfusion pressure. Dotta et al. have demonstrated that cerebral blood flow can decrease following primary closure of a large CDH (16). This underscores the importance of preventing IAH/ACS when making closure decisions.

## Conclusion

Intraoperative BP monitoring is a simple and effective objective tool for guiding abdominal closure management in neonates with CDH, especially those on ECMO. We propose its routine integration into surgical practice to standardize decisions regarding DAC. This approach minimizes the variability and delay inherent in subjective assessment, thereby helping to prevent abdominal compartment syndrome, enhancing patient safety, and potentially improving outcomes in this high-risk population.

## Patient's mother perspective

“As parents, our journey through our child's CDH diagnosis and treatment was marked by profound uncertainty. The prenatal diagnosis filled us with anxiety, which only deepened after birth when his condition rapidly deteriorated, necessitating ECMO support. In those critical moments, we placed our complete trust in the medical team. Witnessing our child successfully wean from ECMO, undergo final closure, and gradually recover has filled us with immense gratitude. This experience taught us that in caring for the most fragile lives, clear communication is as vital as advanced technology. We sincerely hope that sharing our story may offer hope and insight to other families facing similar challenges.”

## Data Availability

The raw data supporting the conclusions of this article will be made available by the authors, without undue reservation.
